# Isolated Central Epiretinal Membrane: A Rare Complication of Fovea-Sparing Internal Limiting Membrane Peeling Technique

**DOI:** 10.1155/2021/6654604

**Published:** 2021-04-14

**Authors:** Yen-Chih Chen, San-Ni Chen

**Affiliations:** ^1^Department of Ophthalmology, Changhua Christian Hospital, Changhua, Taiwan; ^2^Department of Ophthalmology, Yunlin Christian Hospital, Xiluo, Yunlin, Taiwan; ^3^Department of Optometry, Central Taiwan University of Science and Technology, Taichung, Taiwan; ^4^School of Medicine, Chung-Shan Medical University, Taichung, Taiwan; ^5^School of Medicine, Kaohsiung Medical University, Kaohsiung, Taiwan; ^6^Department of Optometry, Da-Yeh University, Changhua, Taiwan

## Abstract

**Purpose:**

To report a rare complication presenting as an isolated central epiretinal membrane (ERM) related to fovea-sparing internal limiting membrane (ILM) peeling technique.

**Methods:**

Five patients who received fovea-sparing ILM peeling were enrolled. Postoperatively, an isolated central ERM developed. Optical coherence tomography (OCT) was used to evaluate the serial anatomic change.

**Results:**

Among the five included patients, one patient had high myopia with foveoschisis, two patients had vitreomacular traction, and two patients had proliferative diabetic retinopathy with tractional retinal detachment and a fovea cyst. With an average of 5.80 months, OCT showed the gradual development of the isolated central ERM with severe fovea distortion. Four patients received secondary revision surgery, with improvement of the fovea contour and visual acuity.

**Conclusion:**

The fovea-sparing ILM peeling technique may cause a rare but serious complication as the isolated central ERM, which would cause significant fovea distortion as well as visual deterioration. Timely detection and intervention is recommended to prevent further visual loss. This trial is registered with NCT04445142.

## 1. Introduction

Internal limiting membrane (ILM) peeling is considered as a fundamental step in vitreomacular surgery, including epiretinal membrane (ERM), lamellar macular holes, full-thickness macular holes (MH), and even for diseases such as diabetic macular edema and retinal vein occlusion [1–4]. Recently, a fovea-sparing ILM peeling method was introduced, which aims to remove a ring of the ILM around the macula while sparing a small portion of the ILM tissue over the fovea. It was found to be safer and to have a better functional outcome than complete ILM peeling, especially for cases with myopic foveoschisis [5] and small size full-thickness MH [6, 7].

However, it is known that ERM may occur without ILM peeling. For patients who underwent fovea-sparing ILM peeling, the central residual ILM may easily cause secondary ERM formation exclusively on the foveal tissue. So far, the number of studies reporting complications regarding fovea-sparing ILM peeling is limited. Here, we report a case series of patients who had undergone fovea-sparing ILM peeling and later developed an isolated central ERM associated with foveal distortion and visual acuity impairment.

## 2. Materials and Methods

This was a retrospective case series. Patients who underwent fovea-sparing ILM peeling from January 2019 to July 2020 who developed an isolated central ERM were included. All patients had regular follow-up at our clinic and had undergone ophthalmic examination including visual acuity, slit lamp biomicroscopy, and fundus ophthalmoscopy at each visit. The surgical technique of fovea-sparing ILM peeling was performed as previously described [7]. Spectral domain optical coherence tomography (OCT) (Heidelberg Spectralis; Heidelberg Engineering, Heidelberg, Germany) was used to examine the postoperative anatomy of the retina. The first and second operations were performed by a single and experienced surgeon (SN Chen). The study had the approval of the Institutional Review Board of Changhua Christian Hospital and was performed in accordance with the World Medical Association's Declaration of Helsinki.

## 3. Statistical Analysis

The statistical analysis was performed using MedCalc software version 19.6.1 (MedCalc Software, Mariakerke, Belgium). The best-corrected visual acuity (BCVA) was converted to the logarithm of the minimal angle of resolution (logMAR) equivalent for statistical analysis. For the four patients receiving revision surgery, Wilcoxon sign rank test was performed to compare the differences of BCVA and central fovea thickness (CFT) on OCT before and after revision surgery. In all analyses, a *p* value < 0.05 was considered statistically significant.

## 4. Results

During the inclusion period, 46 patients underwent fovea-sparing ILM peeling surgery. During the follow-up period, five patients were noted to have developed isolated central ERM. The demographics and clinical features of the five patients are reported in Table 1.

**Figure 1:**
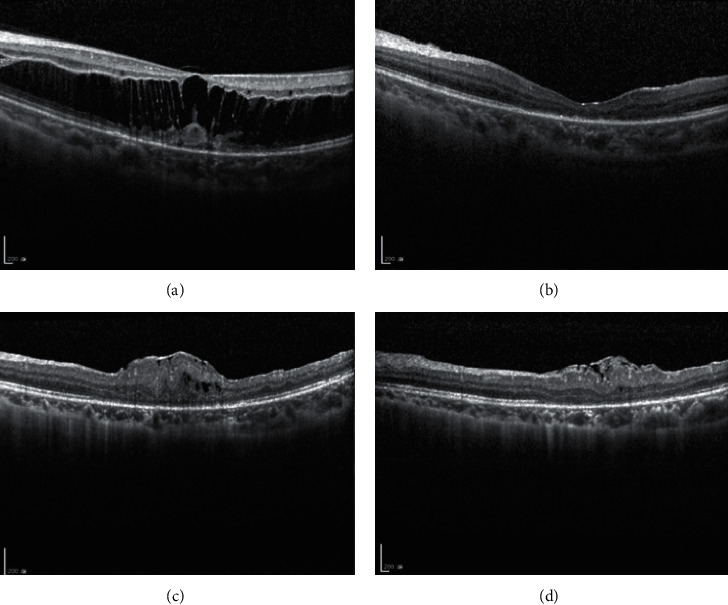
Example of the isolated central epiretinal membrane (ERM) development after fovea-sparing internal limiting membrane (ILM) peeling technique in a case with myopic foveoschisis (case 2). (*a*) A 10-year-old boy had pathologic myopia and macular foveoschisis of the left eye. The best-corrected visual acuity of his left eye was 20/80. He received vitrectomy with fovea-sparing ILM peeling. (*b*) Postoperatively, the foveoschisis improved. (*c*) However, 3 months later, an isolated central fovea ERM gradually developed, and optical coherence tomography (OCT) demonstrated severe contraction of the ERM with bulging fovea contour. The central fovea thickness (CFT) was 404 *µ*m, and his visual acuity deteriorated to 20/100. (*d*) 1 month later, follow-up OCT showed spontaneous peeling of the central ERM with decreased fovea distortion. The CFT improved to 204 *µ*m, and the second revision surgery was therefore postponed.

There were three high myopia patients; one patient developed foveoschisis (Figure 1), and two patients developed vitreomacular traction (VMT).

**Figure 2:**
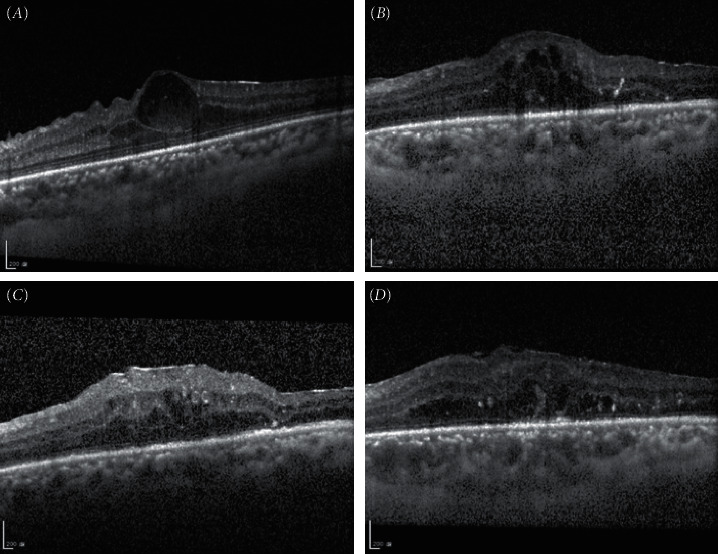
Example of the isolated central epiretinal membrane (ERM) development after fovea-sparing internal limiting membrane (ILM) peeling technique in a case with proliferative diabetic retinopathy with a fovea cyst (case 5). (*A*) A 39-year-old male patient had proliferative diabetic retinopathy and a fovea cyst with very thin fovea tissue. He received vitrectomy due to persistent macular edema despite several antivascular endothelial growth factor injections. During vitrectomy, concerning the very thin fovea tissue, we performed fovea-sparing ILM peeling to prevent inadvertent avulsion of fovea tissue. However, after the surgery, optical coherence tomography (OCT) showed the development of isolated central ERM formation with progression 1 month (*B*) and 3 months (*C*) later. The central fovea thickness (CFT) was 569 *µ*m, and his visual acuity deteriorated to 20/400. We arranged second surgery to remove the central ERM and residual fovea ILM. (*D*) After second surgery, OCT showed improvement in fovea contour. Three months after the revision surgery, the CFT improved to 440 *µ*m, and his visual acuity was 20/100.

Two patients had proliferative diabetic retinopathy; one patient developed tractional retinal detachment, and one patient developed a foveal cyst (Figure 2).

All patients underwent vitrectomy followed by fovea-sparing ILM peeling to prevent damage to the foveal tissue during membrane peeling. For all patients, triamcinolone acetonide was routinely used to stain the vitreous during vitrectomy, and 0.05% indocyanine green (ICG) was used to stain the ILM. Postoperatively, serial OCT revealed the formation of an isolated central ERM with severe contraction at an average of 5.80 ± 2.68 months following surgery. The central ERM caused severe foveal distortion and visual impairment. Four patients underwent revision surgery to remove the central ERM, while one patient (case 2) experienced spontaneous peeling of the central ERM, which was detected on follow-up OCT. For the four patients receiving revision surgery, postoperative mean CFT on OCT, although nonstatistically significant, showed marked improvement (from 473.00 ± 73.47 *µ*m to 360.50 ± 82.45 *µ*m, *p*=0.06). However, the mean BCVA did not improve significantly (from 0.88 ± 0.55 to 0.48 ± 0.39 in logMAR, *p*=0.20).

## 5. Discussion

Fovea-sparing ILM peeling was recently introduced as a safer surgical alternative in cases wherein MH are present or there is impending macular hole formation as it has a lower rate of secondary macular hole formation or foveal thinning [5–7]. According to the authors, the rationale for sparing the ILM is to preserve the delicate anatomy of the fovea, thereby avoiding further loss of the foveal tissue. However, in this case series, we observed a unique pattern of isolated central ERM formation on the residual central foveal ILM in patients who underwent fovea-sparing ILM peeling. The isolated central ERM caused marked foveal distortion on OCT as well as significant visual impairment.

Sparing the foveal ILM has the theoretical advantage of avoiding iatrogenic damage and preserving the end processes of the Müller cells, which are the main structural elements in the physiology of the cones in this area. In addition to a better anatomic outcome, fovea-sparing ILM peeling was reported to have better retinal sensitivity on microperimetry compared with conventional complete ILM peeling in patients with myopic macular retinoschisis [8], macular pucker [9], degenerative lamellar macular hole [10], and full-thickness macular hole [11]. However, glial cells proliferating over the residual central ILM that cause the formation and contraction of the ERM may cause visual impairment after fovea-sparing ILM peeling. An ERM is an avascular and fibrocellular membrane that proliferates on the inner surface of the retina. The ILM is known as a scaffold for cell proliferation and secondary ERM formation. It was reported in previous literature that recurrent ERM was more often observed in cases without ILM peeling [12–14].

The complication of a secondary central ERM resulting from fovea-sparing ILM peeling is rarely reported. In the study by Russo et al. [9], the authors reported that three out of 19 (15.7%) macular pucker patients who underwent fovea-sparing ILM peeling developed recurrent ERM requiring revision surgery to regain vision. However, the authors did not report the unique pattern of isolated central ERM on OCT. From our report, 46 patients underwent fovea-sparing ILM peeling during the inclusion period, and five of these patients developed an isolated central ERM, with an overall occurrence rate of 10.9%. It is speculated that since the parafoveal ILM had been removed, the residual central ILM may be the primary site for fibrocellular membrane proliferation. The centripetal contraction of the membrane would cause severe foveal distortion on follow-up serial OCT.

In our case series, there were two high myopia patients with VMT and one patient with myopic foveoschisis. It is recognized that a secondary macular hole is frequently seen in cases with myopic foveoschisis due to the extremely thin foveolar tissue [15]. To prevent the development of a postoperative macular hole, fovea-sparing ILM peeling was performed. Although the foveoschisis significantly improved on immediate postoperative OCT, an isolated central ERM eventually developed after an average of six months.

From our report, one of our case of myopic foveoschisis was a 10-year-old child. Pediatric ERMs are most commonly associated with trauma and uveitis [16]. We therefore speculated that ocular surgery itself causes surgical trauma, and the marked postoperative inflammation may hasten the development of an isolated central ERM. Although the revision surgery would improve the foveal contour and visual acuity, we observed that the spontaneous peeling of the ERM was accompanied by an improvement in visual acuity in that boy. Spontaneous ERM peeling in young patients is rare. It is believed that when the contracting forces of the ERM are stronger than its adhesions to the retina, the membrane may separate spontaneously [17]. This phenomenon also implies that the isolated central ERM in young patients would develop and contract to a greater degree with time. However, we are uncertain about the incidence of spontaneous ERM peeling without revision surgery. The timing of the revision surgery must be further investigated in the future.

On the other hand, two patients in our case series had diabetic retinopathy; one patient developed tractional retinal detachment, and one patient developed a foveal cyst. So far, there have been no studies on using fovea-sparing ILM peeling in those with diabetic retinopathy. Theoretically, peeling ILM over the fovea may carry a higher risk of postoperative macular hole formation in patients with tractional retinal detachment and foveal cysts, owing to the very thin foveolar tissue. Therefore, we chose to spare the central foveal ILM in these patients. Unfortunately, an isolated central ERM developed postoperatively in both patients. In proliferative diabetic retinopathy, excessive cytokines or growth factors secondary to changes in vascular permeability and retinal ischemia would more easily stimulate glial proliferation and cause the formation of a central ERM [18]. The formation of an isolated central ERM may predispose these patients to diabetic macular edema which will worsen visual acuity. Therefore, caution should be observed when using this technique in patients with diabetic retinopathy.

In our report, four patients required revision surgery after an average follow-up of 5.8 months. By removing the central ERM and foveal ILM, we observed that the foveal thickness as well as visual acuity although not statistically significantly but both showed marked improvement. To improve on this study, longer follow-up periods and a larger number of cases are necessary to evaluate the possible long-term effects of an isolated central ERM and the role of revision surgery on foveal contour and visual acuity.

To the best of our knowledge, this is the first report describing the rare complication of an isolated central ERM on serial OCT in patients who underwent fovea-sparing ILM peeling. However, there are several limitations in our report. First, owing to the rarity of the complication, the number of patients included in the study was small. The five patients in our case series are heterogenous, and the results of the analysis may not be conclusive. However, an analysis comparing only either idiopathic ERM or PDR-related ERM would be difficult. Larger studies and more cases are needed to make a subgroup analysis and analyze the possible risk factors contributing to this complication, respectively. Second, although we report the rare complication of an isolated central ERM from our patients who underwent fovea-sparing ILM peeling, it is still uncertain if this technique would directly contribute to the formation of an isolated central ERM. Recurrent and residual ERM are commonly observed after conventional ILM peeling. However, the unique pattern of an isolated central ERM is rarely observed. Since the case number is relatively small, larger cohort studies would be needed to prove this assumption. Third, some of our patients already had an ERM prior to undergoing fovea-sparing ILM peeling. During fovea-sparing ILM peeling, both the ERM and ILM at the parafoveal area were removed. However, it is uncertain whether the ERM recently developed or was a recurrence from the peripheral areas. Closer postoperative follow-up visits with OCT may clarify this.

## 6. Conclusion

In conclusion, although the fovea-sparing ILM peeling technique is theoretically more beneficial than complete ILM peeling, we had demonstrated the rare complication as formation of an isolated central ERM. The central ERM would contract progressively and cause significant visual impairment. Most patients required revision surgery to regain foveal contour and visual acuity. Timely detection and intervention is recommended.

## Figures and Tables

**Table 1:** Demographic data of patients.

Case/age/sex/eye	Diagnosis	Time interval of ERM development (months)	Best-corrected visual acuity (logMAR)			Central fovea thickness (um)		
			Before 1^st^ surgery	Before revision surgery	After revision surgery	Before 1^st^ surgery	Before revision surgery	After revision surgery
1/47/F/OD	High myopia with VMT	6	0.2	0.4	0.1	372	390	360
2/10/M/OS	High myopia with foveoschisis	4	1.6	0.7	0.5	428	404	204^*∗*^
3/54/F/OD	High myopia with VMT	10	0.7	0.5	0.3	532	468	395
4/38/M/OD	PDR + TRD	3	0.5	1.6	0.5	592	465	247
5/40/M/OD	PDR + fovea cyst	6	0.2	1	1	508	569	440

^*∗*^No revision surgery due to spontaneous membrane peeling. F: female; M: male; PDR: proliferative diabetic retinopathy; VMT: vitreomacular traction; TRD: tractional retinal detachment.

## Data Availability

The data used to support the findings of this study are available from the corresponding author upon request.
